# Supplementary material for: Tetranuclear Copper(I) and Silver(I) Pyrazolate Adducts with 1,1′-Dimethyl-2,2’-bibenzimidazole: Influence of Structure on Photophysics
Source: Molecules. 2023 Jan 25;28(3):1189. doi: 10.3390/molecules28031189 (PMC9920877; doi:10.3390/molecules28031189)
Supplement: Supplementary file 1 [file molecules-28-01189-s001.zip › molecules-2161440-supplementary.pdf]

Supporting Information for

Tetranuclear copper(I) and silver(I) pyrazolate adducts with 1,1′-Dimethyl-2,2'-bibenzimidazole: influence of structure on photophysics

Gleb B. Yakovlev[1],Aleksei A. Titov*[1], Alexander F. Smol'yakov[1], Andrey Yu. Chernyadyev[2], Oleg A. Filippov[1], Elena S. Shubina[1]

[1] A. N. Nesmeyanov Institute of Organoelement Compounds, Russian Academy of Sciences, Vavilov Str., 28,

119991 Moscow, Russia; gleb240602@mail.ru (G.B.Y.); tit@ineos.ac.ru (A.A.T.); h-bond@ineos.ac.ru (O.A.F.);

rengenhik@gmail.com (A.F.S.), shu@ineos.ac.ru (E.S.S.);

[2] A. N. Frumkin Institute of Physical Chemistry and Electrochemistry, Russian Academy of Sciences,

Leninsky prosp. 31/4, 199071 Moscow, Russia; chernyadyev@mail.ru (A. Y. C.);

* Correspondence: tit@ineos.ac.ru (A.A.T.)

Table S1. Crystal data, data collection, and structure refinement parameters for 1-4

Complex	1	2	3	5
Empirical formula	$C_{52}H_{32}Cu_4F_{24}N_{16}$	$C_{36}H_{18}Cu_4F_{24}N_{12}$	$C_{52}H_{32}Ag_4F_{24}N_{16}$	$C_{36}H_{18}Ag_4F_{24}N_{12}$
Formula weight	1591.09	1328.78	1768.41	1506.1
Temperature/K	100	100	100	120
Crystal system	tetragonal	monoclinic	monoclinic	monoclinic
Space group	I-4	C2/c	Cc	Cc
a/Å	12.1309(4)	34.6187(7)	23.491(2)	12.460(2)
b/Å	12.1309(4)	12.5461(3)	12.2873(11)	23.856(4)
c/Å	19.7184(12)	21.1280(4)	22.922(2)	15.954(3)
α/°	90	90	90	90
β/°	90	96.2900(10)	115.291(5)	92.364(4)
γ/°	90	90	90	90
Volume/Å^3	2901.7(3)	9121.3(3)	5982.0(10)	4738.3(13)
Z	2	8	4	4
ρ_{calc}g/cm^3	1.821	1.935	1.964	2.111
μ/mm^{-1}	1.578	1.984	1.418	1.767
F(000)	1576	5200	3440	2888
Crystal size/mm^3	0.16 × 0.15 × 0.14	0.22 × 0.14 × 0.09	0.18 × 0.14 × 0.09	0.25 × 0.18 × 0.14
Radiation	MoKα (λ = 0.71073)	MoKα (λ = 0.71073)	MoKα (λ = 0.71073)	MoKα (λ = 0.71073)
2Θ range for data collection/°	3.942 to 51.966	3.456 to 59.288	3.83 to 51.998	3.69 to 51.998
Reflections collected	13361	63273	17325	17279
Independent reflections	2845 [R_{int} = 0.0688, R_{sigma} = 0.0518]	12863 [R_{int} = 0.0640, R_{sigma} = 0.0531]	17325 [R_{int} = 0.0640, R_{sigma} = 0.1215]	8393 [R_{int} = 0.0503, R_{sigma} = 0.0704]
Data/restraints/parameters	2845/18/219	12863/0/687	17325/164/870	8393/2/688
Goodness-of-fit on F^2	1.116	1.031	1.039	1.031
Final R indexes [I>=2σ (I)]	R_1 = 0.1108, wR_2 = 0.2922	R_1 = 0.0452, wR_2 = 0.1054	R_1 = 0.0773, wR_2 = 0.1879	R_1 = 0.0519, wR_2 = 0.1345
Final R indexes [all data]	R_1 = 0.1114, wR_2 = 0.2925	R_1 = 0.0688, wR_2 = 0.1161	R_1 = 0.0984, wR_2 = 0.2033	R_1 = 0.0572, wR_2 = 0.1422
Largest diff. peak/hole / e Å^{-3}	1.51/-1.82	1.82/-1.03	2.76/-1.58	2.08/-1.80

	298 K	77 K
1		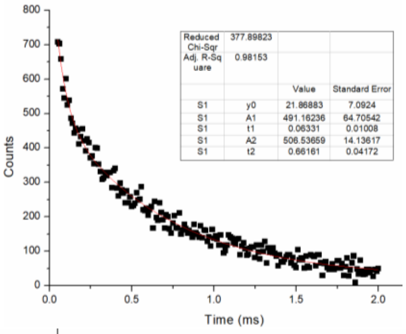
2		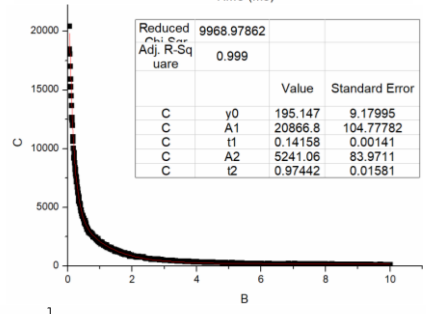
3		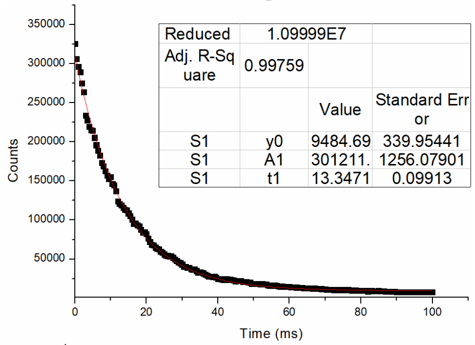
4	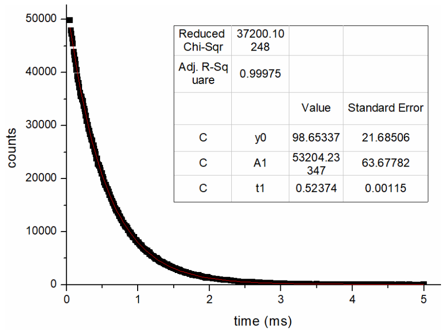	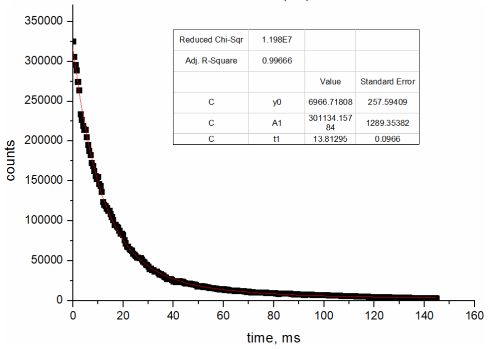

Figure S1. Phosphorescence decays of complexes 1-4 in the solid state at 298 K and 77 K.

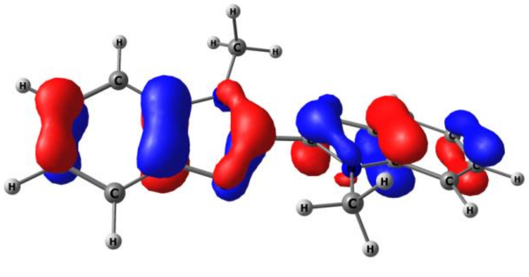

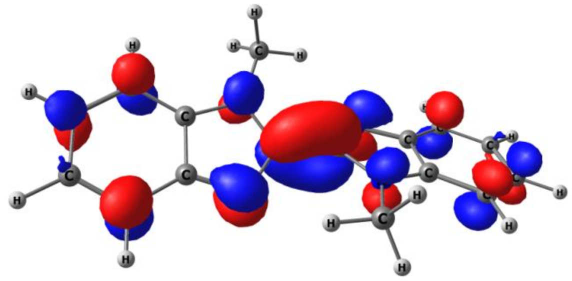

S1 HONTO (E=37799.2 cm^{-1}, 264.6 nm, f=1.3104)

LUNTO

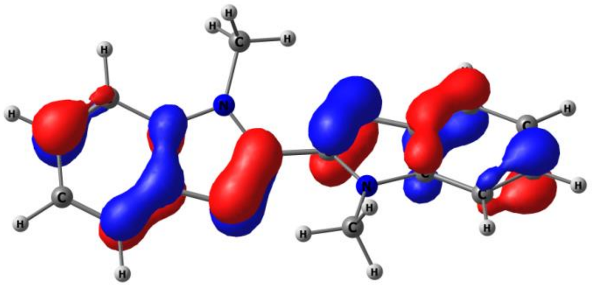

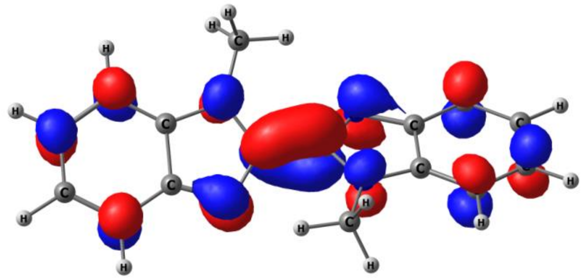

T1 HONTO E=29735.5 cm^{-1}, 336.3 nm

LUNTO

Figure S2. Natural transition orbitals HONTO (left) and LUNTO (right) for $S_0 \rightarrow S_1$ excitation of anty-geometry of **L** as isosurface at 0.05 a.u.

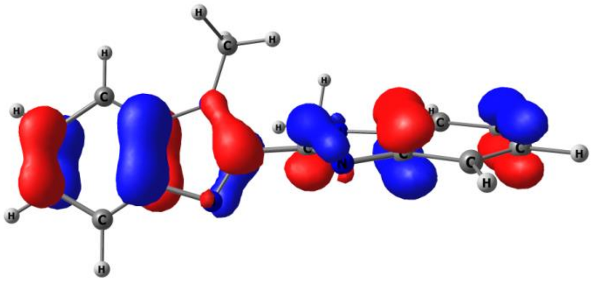

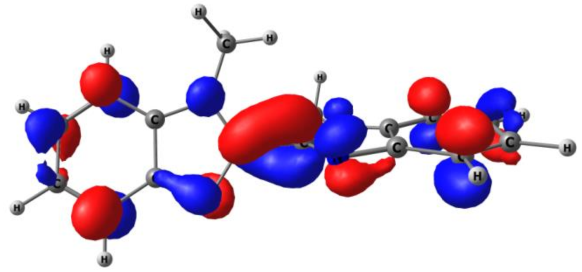

S1 HONTO (E=40328.8 cm^{-1}, 248.0 nm, f=0.9691)

LUNTO

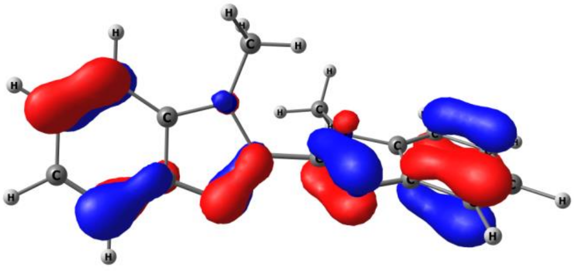

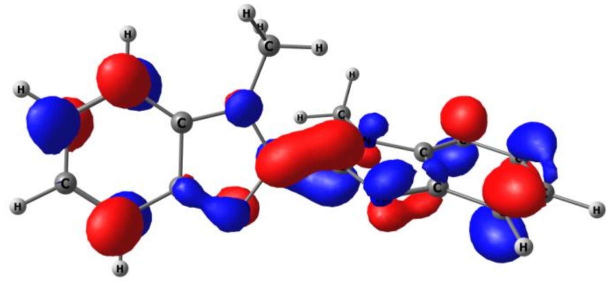

T1 HONTO E=30987.7 cm^{-1}, 322.7 nm

LUNTO

Figure S3. Natural transition orbitals HONTO (left) and LUNTO (right) for $S_0 \rightarrow S_1$ excitation of syn-geometry of **L** as isosurface at 0.05 a.u.

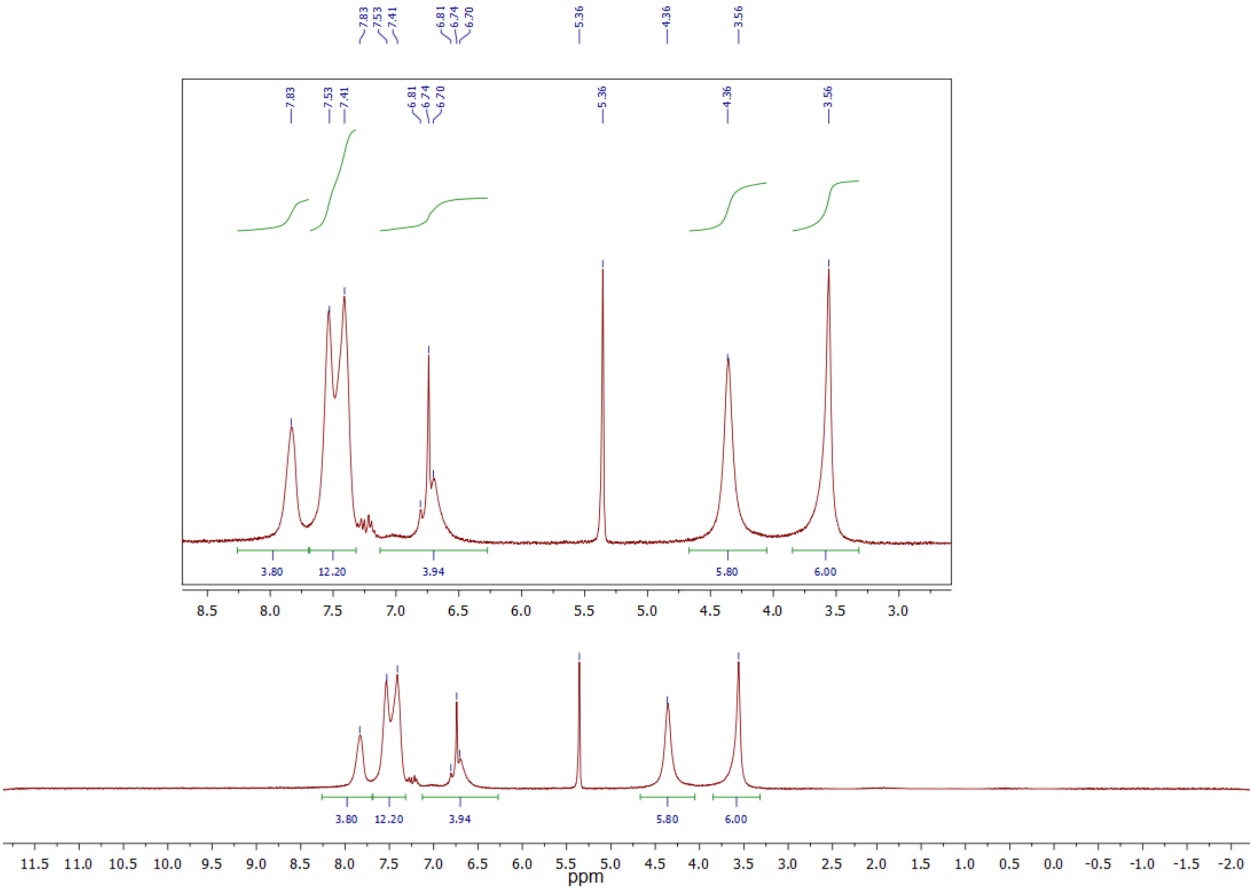

Figure S4. ^{1}H NMR spectrum of **1** in CD_2Cl_2.

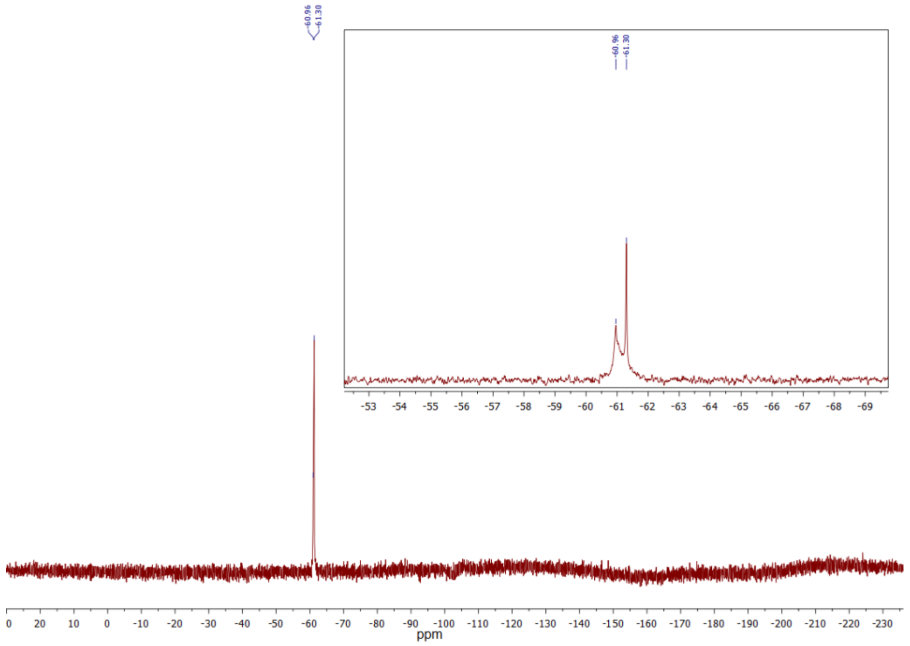

Figure S5. [1]F NMR spectrum of **1** in CD_2Cl_2.

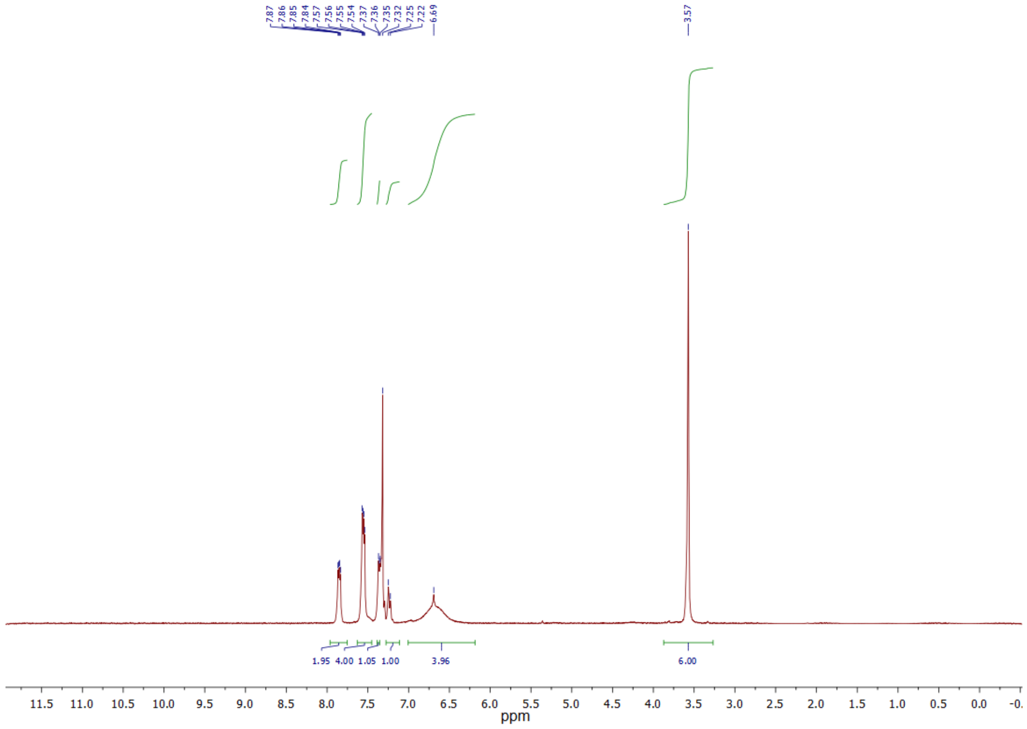

Figure S6. ^{1}H NMR spectrum of **2** in $CDCl_3$.

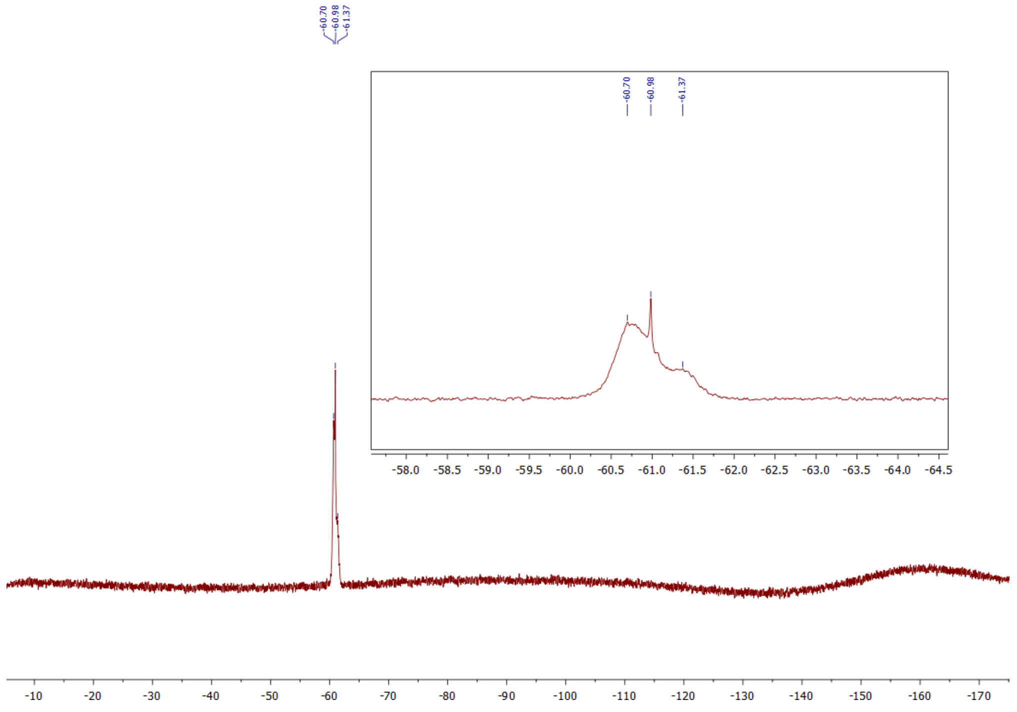

Figure S7. ^{19}F NMR spectrum of **2** in $CDCl_3$.

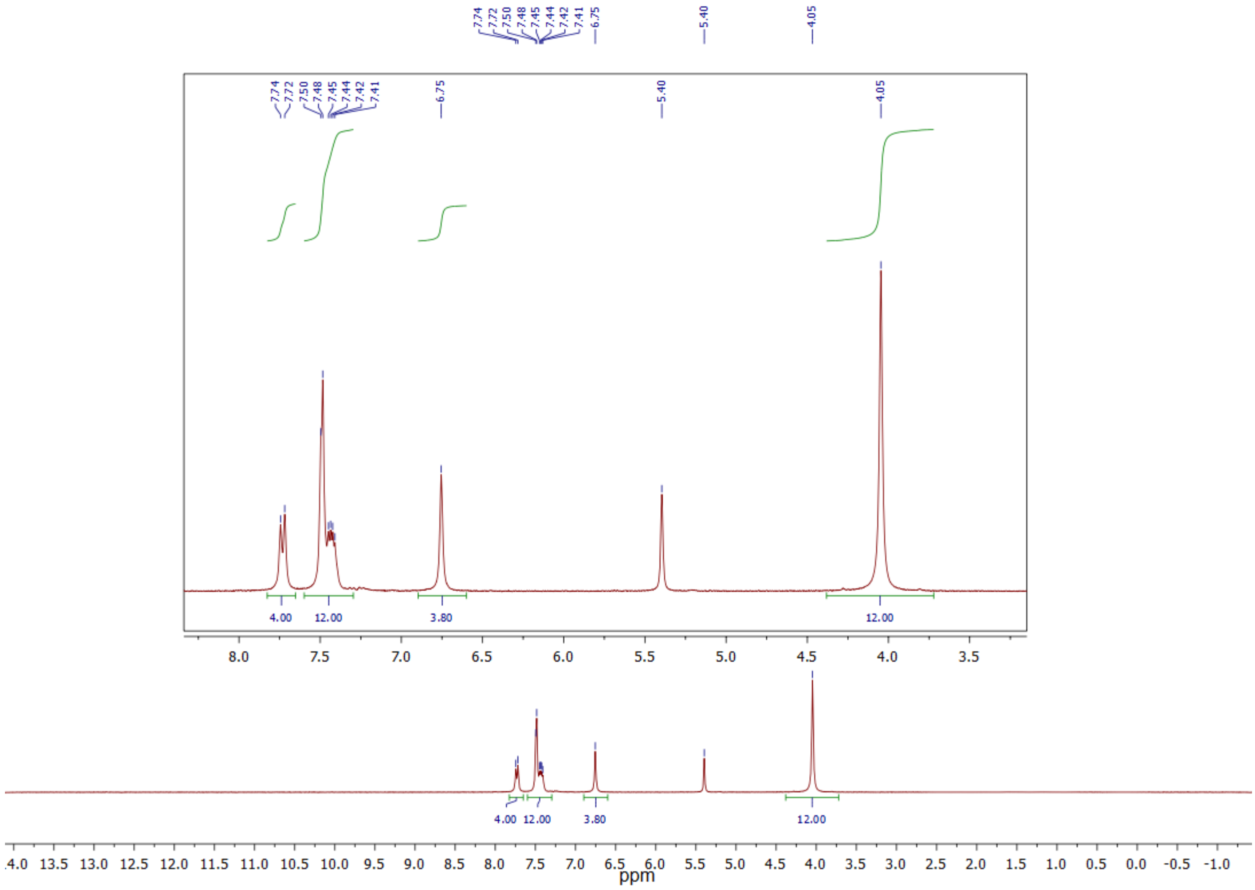

Figure S8. ^{1}H NMR spectrum of **3** in CD_2Cl_2.

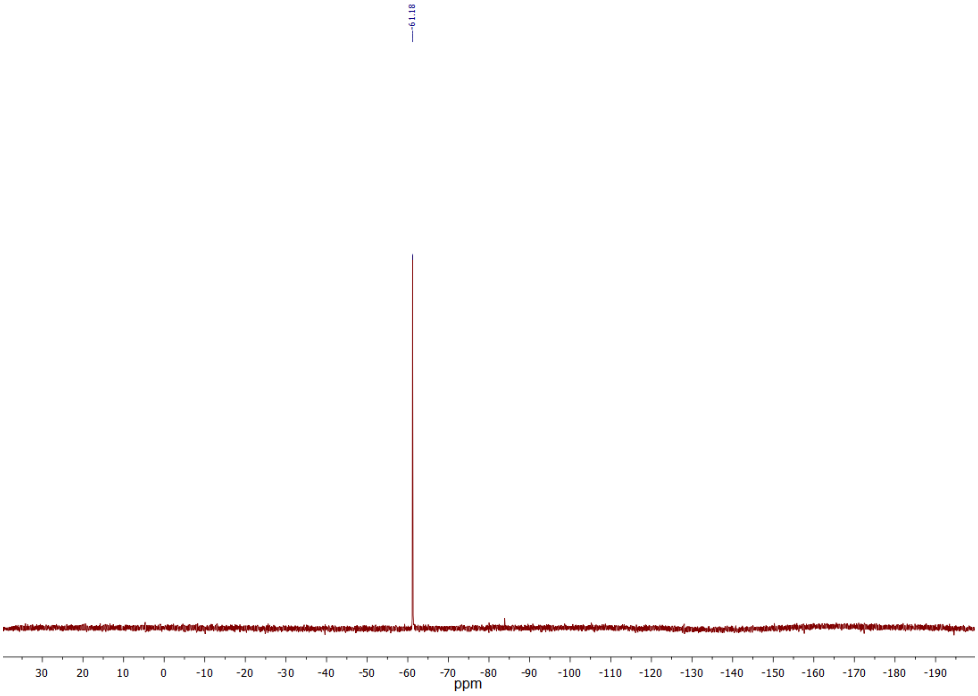

Figure S9. ^{19}F NMR spectrum of **3** in CD_2Cl_2.

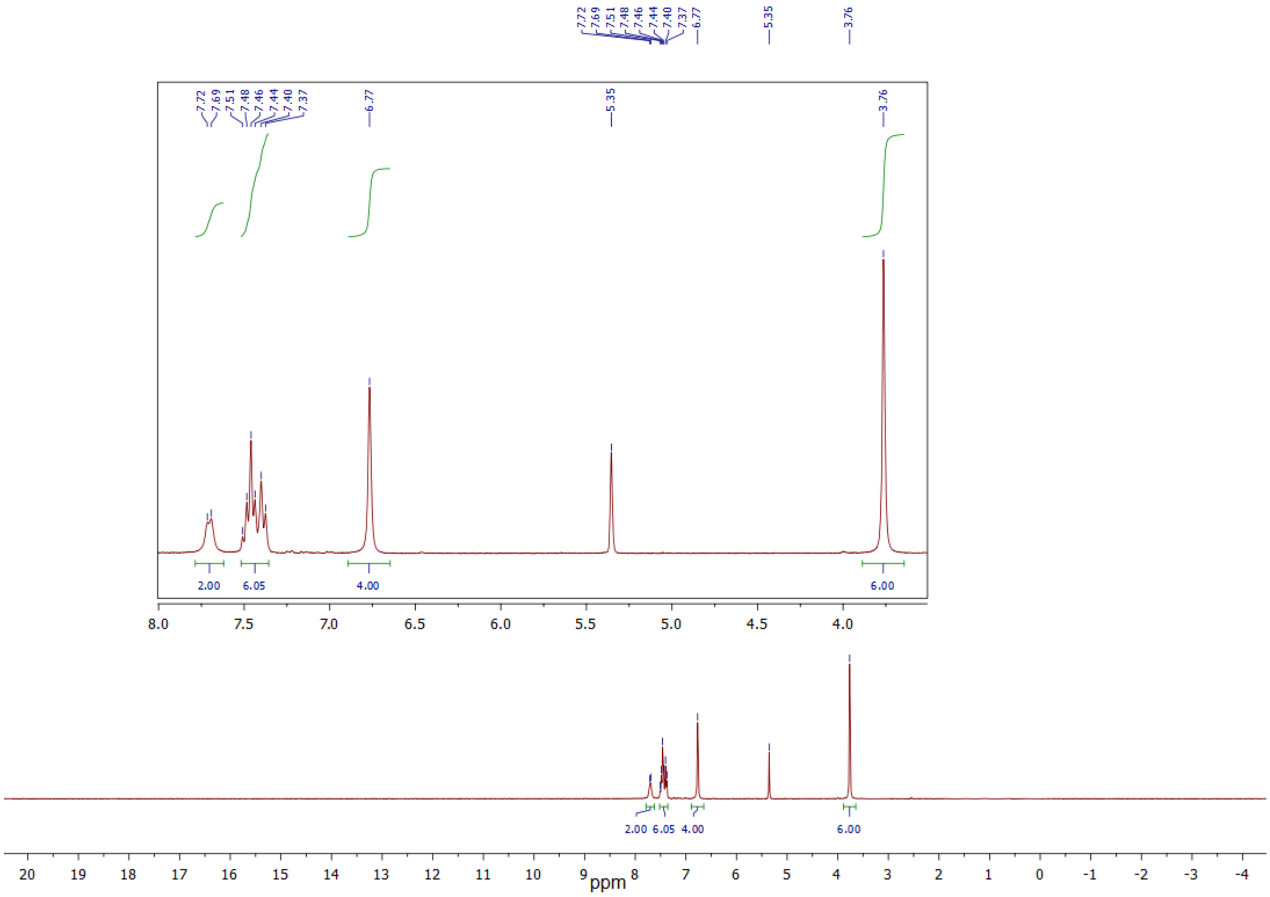

Figure S10. ¹H NMR spectrum of **4** in CD_2Cl_2.

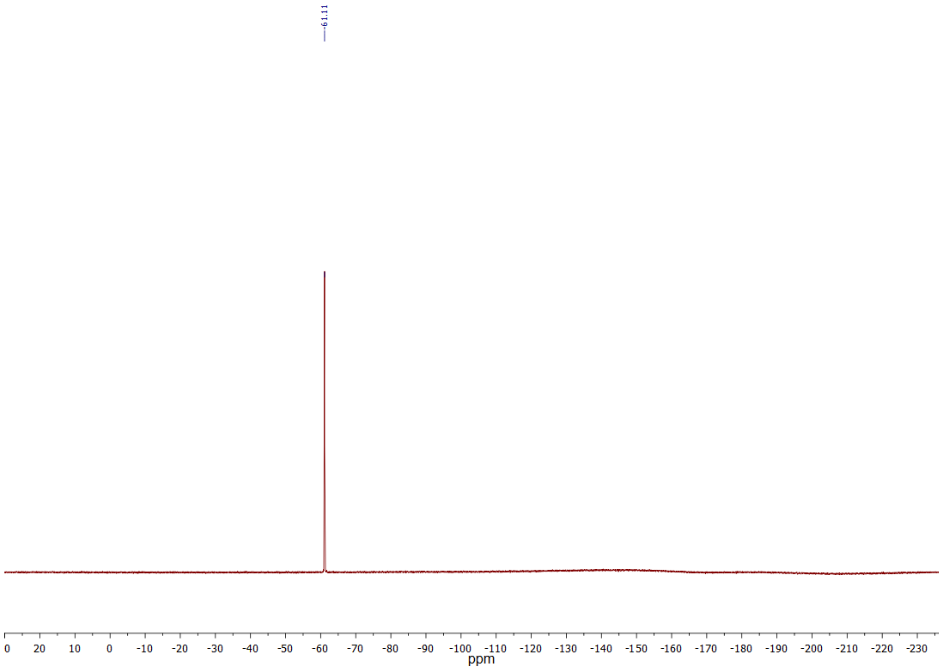

Figure S 11. ¹⁹F NMR spectrum of **4** in CD_2Cl_2.

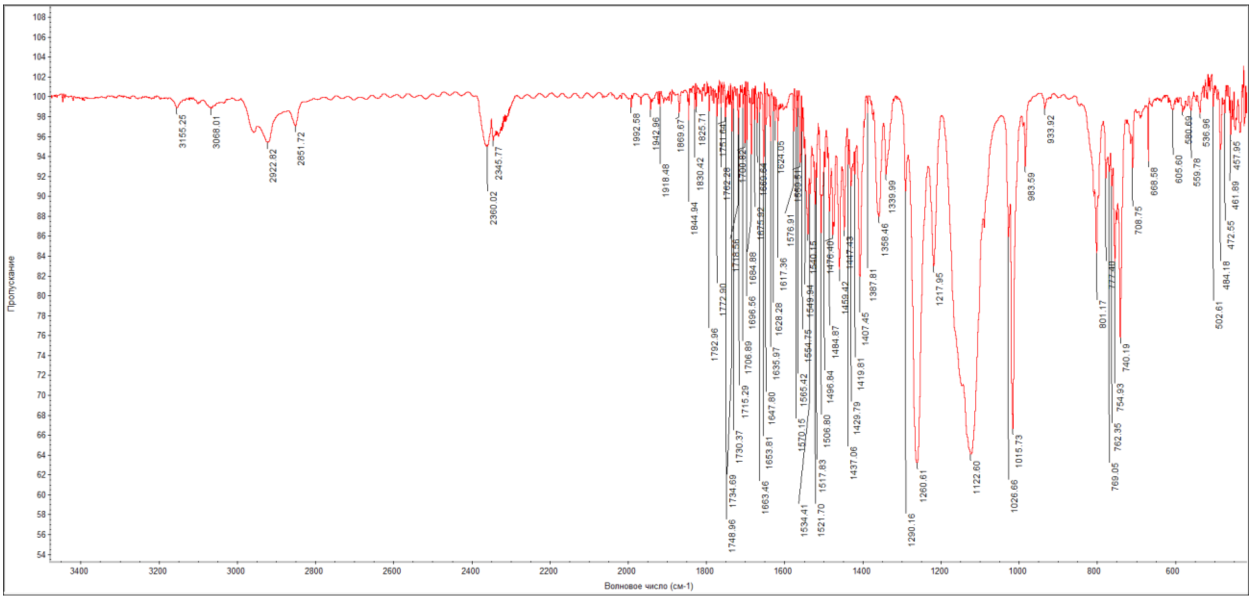

Figure S12. IR spectrum for complex **1** (KBr).

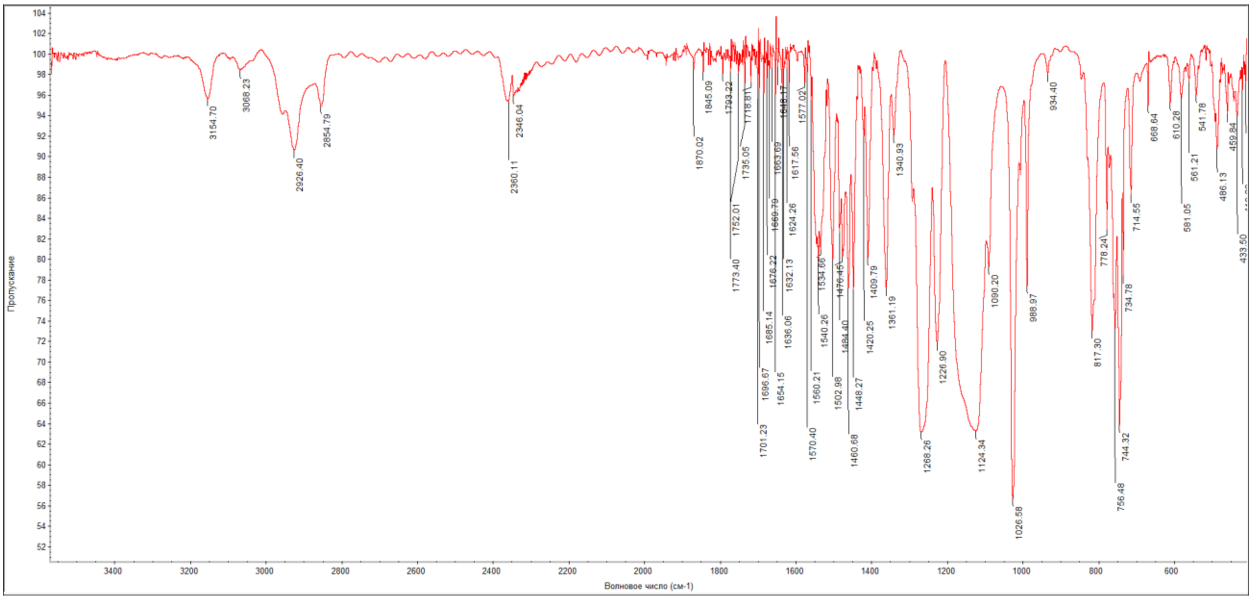

Figure S 13. IR spectrum for complex **2** (KBr).

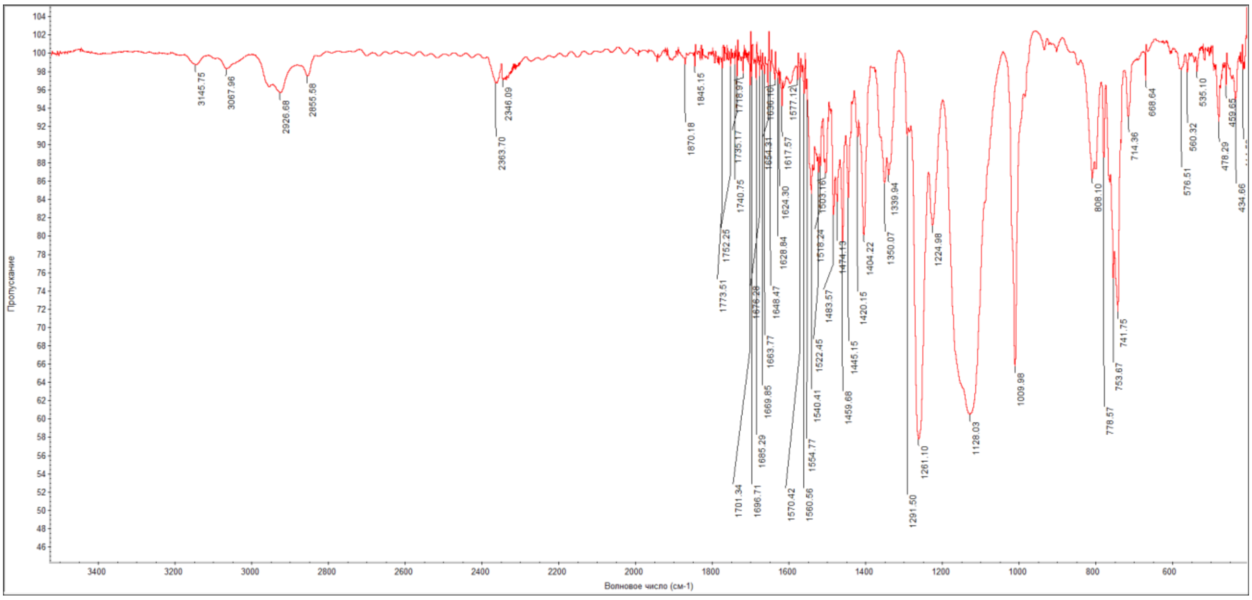

Figure S 14. IR spectrum for complex **3** (KBr).

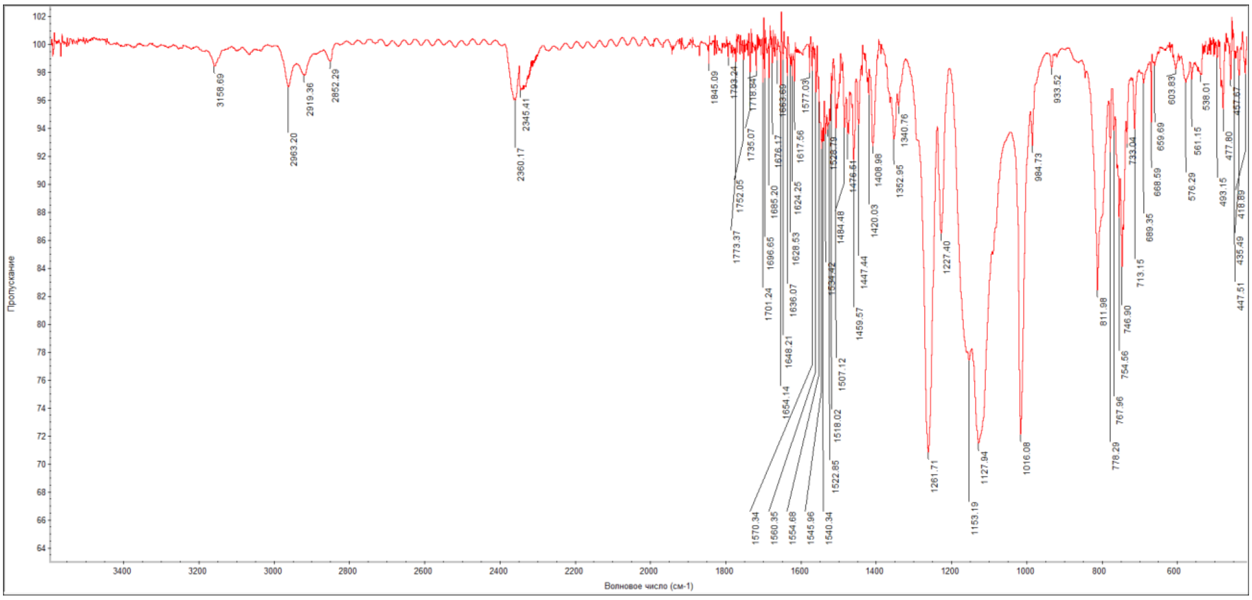

Figure S 15. IR spectrum for complex **4** (KBr).

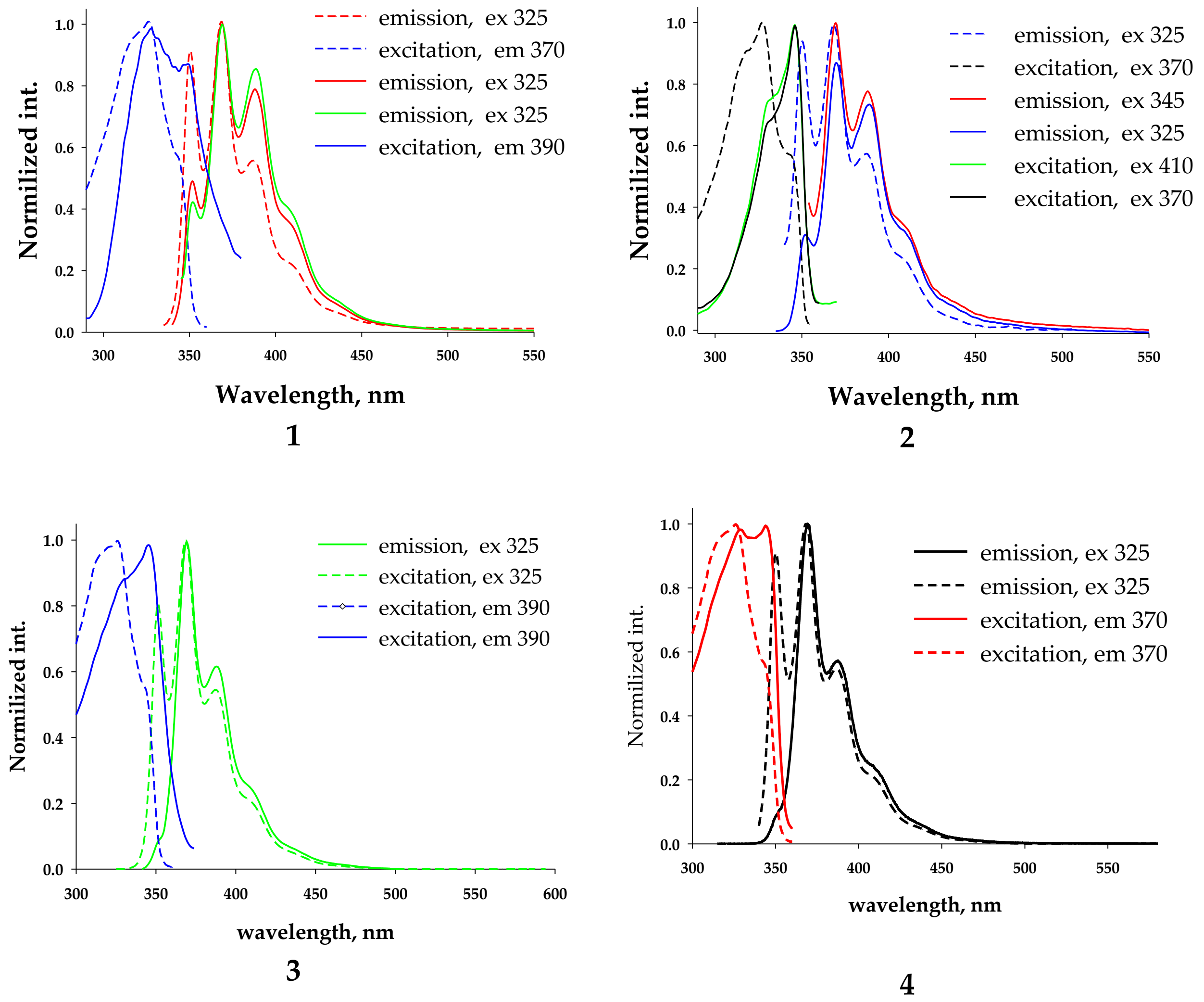

Figure S16. Emission an excitation spectra of complexes 1-4 at different concentrations (298 K): $c=1\cdot10^{-5}$ M (dashed lines) and $c=1.2\cdot10^{-2}$ M (solid lines).

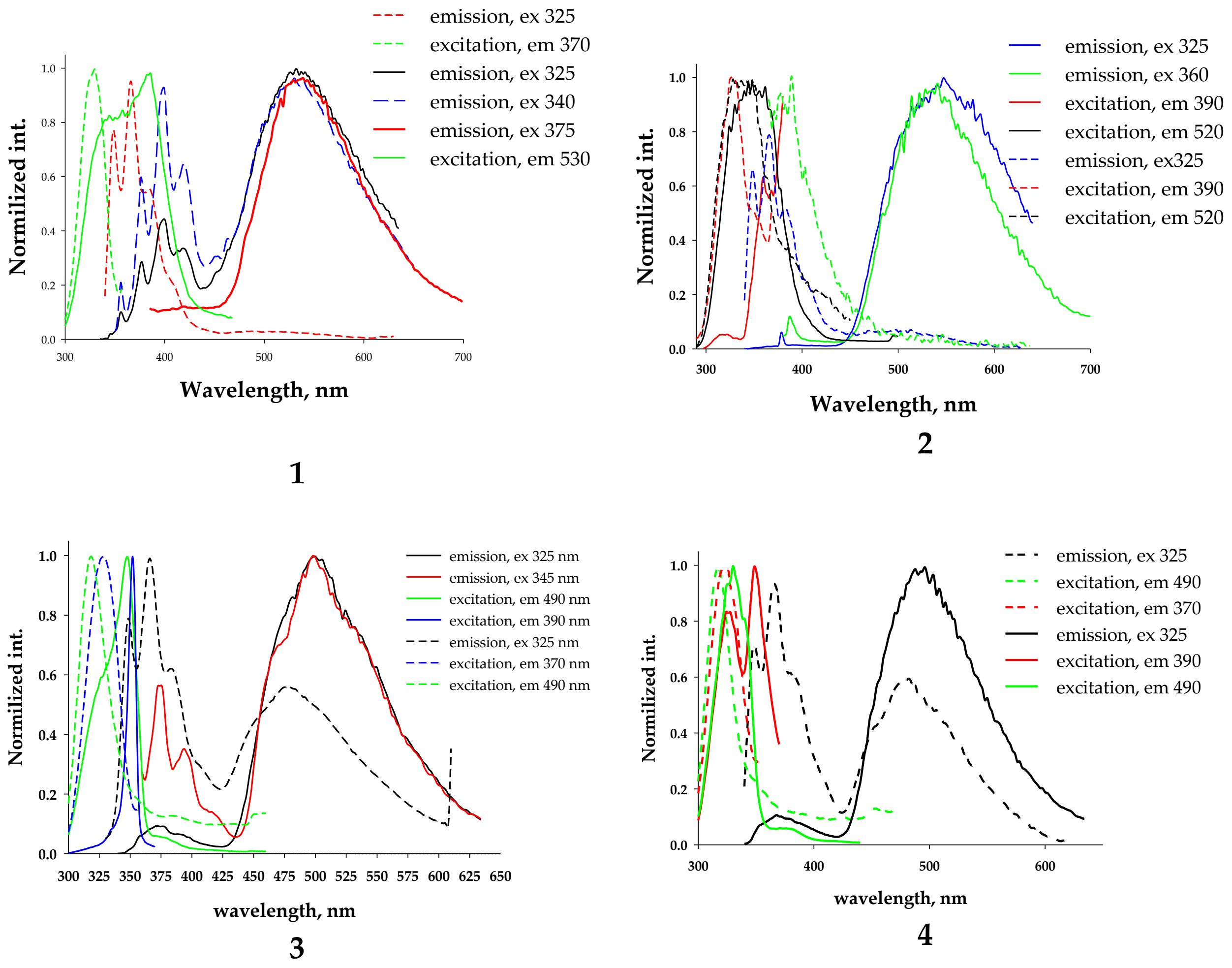

Figure S17. Emission an excitation spectra of complexes 1-4 at different concentrations (77 K, frozen solution): $c=1\cdot10^{-5}$ M (dashed lines) and $c=1.2\cdot10^{-2}$ M (solid lines).

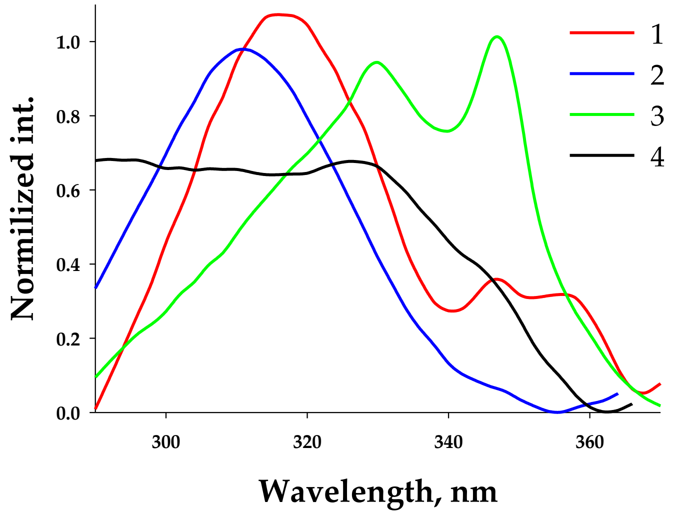

Figure S18. Excitation spectra of complexes, λ_{em}=390 nm

Table S2. Characteristics of $S_0 \rightarrow S_x$ vertical transitions and fragments contributions to the GS and ES electron density for complex **1**.

				GS (hole)			ES (electron)		
state	E, cm^{-1}	λ, nm	f	M	L	Pz	M	L	Pz
S1	31181.8	320.7	0.0018	80	8	11	9	90	1
S2	31295.0	319.5	0.0017	79	9	12	5	94	1
S3	31792.8	314.5	0.0067	80	8	12	13	86	1
S4	32059.2	311.9	0.0081	80	9	11	9	89	1
S5	32550.4	307.2	0.0024	82	4	13	23	75	2
S6	32602.5	306.7	0.0097	84	3	13	19	80	2
S7	32778.8	305.1	0.0181	83	4	14	23	74	3
S8	33174.2	301.4	0.0082	84	3	13	32	65	3
S9	33466.3	298.8	0.0058	82	5	13	50	46	4
S10	33943.6	294.6	0.0020	83	5	12	61	34	5
S11	34015.7	294.0	0.0016	83	5	13	59	36	5
S12	34234.7	292.1	0.0012	83	3	13	58	37	5
S13	34993.4	285.8	0.0003	85	4	11	78	15	7
S14	35160.0	284.4	0.0003	86	3	11	72	22	6
S15	35439.9	282.2	0.0003	85	4	10	79	14	7
S16	36331.3	275.2	0.0164	88	2	9	75	18	7
S17	36935.6	270.7	0.0348	94	1	4	8	91	1
S18	37153.6	269.2	0.0116	94	2	4	4	96	0
S19	37659.0	265.5	0.1158	70	27	3	13	86	1
S20	37830.9	264.3	0.1555	66	31	2	12	87	1

Table S3 Characteristics of $S_0 \rightarrow T_x$ vertical transitions and fragments contributions to the GS and ES electron density for complex **1**.

			GS (hole)			ES (electron)		
state	E, cm^{-1}	λ, nm	M	L	Pz	M	L	Pz
T1	29416.7	339.9	24	74	2	1	99	1
T2	29529.2	338.6	22	75	2	1	99	0
T3	29597.0	337.9	14	85	1	1	99	1
T4	29609.6	337.7	16	83	1	0	99	1
T5	30751.5	325.2	71	18	11	24	74	2
T6	30946.9	323.1	68	22	10	7	92	1
T7	31237.2	320.1	75	14	11	18	80	2
T8	31467.6	317.8	75	14	11	14	84	2
T9	31885.5	313.6	81	6	13	56	39	5
T10	32179.5	310.8	78	9	13	53	42	5
T11	32269.7	309.9	83	5	12	45	51	4
T12	32518.6	307.5	81	7	12	54	41	5
T13	32820.1	304.7	69	19	11	21	77	2
T14	32942.8	303.6	54	38	8	29	68	3
T15	33121.5	301.9	48	45	7	17	81	2
T16	33336.1	300.0	33	61	6	17	81	2
T17	33509.5	298.4	56	35	9	23	75	2

T18	33564.1	297.9	71	17	12	30	68	3
T19	33943.6	294.6	85	4	11	72	22	6
T20	34117.2	293.1	86	3	11	54	41	5

Table S4. Characteristics of $S_0 \rightarrow S_x$ vertical transitions and fragments contributions to the GS and ES electron density for complex **2**.

				GS (hole)			ES (electron)		
state	E, cm^{-1}	λ, nm	f	M	L	Pz	M	L	Pz
S1	33206.7	301.1	0.0230	80	9	11	3	94	2
S2	34002.8	294.1	0.0148	80	10	10	4	94	2
S3	34972.8	285.9	0.0404	81	9	11	3	94	3
S4	35766.9	279.6	0.0301	80	10	10	3	94	3
S5	37100.0	269.5	0.0114	84	4	12	45	9	46
S6	37872.6	264.0	0.0161	83	6	11	45	12	44
S7	38758.4	258.0	0.0444	82	9	9	22	48	29
S8	38853.7	257.4	0.0158	89	5	6	14	67	19
S9	39090.3	255.8	0.5938	35	60	4	7	82	11
S10	39254.5	254.7	0.1070	80	14	6	5	85	10
S11	39581.9	252.6	0.0790	84	5	11	38	17	45
S12	40563.4	246.5	0.0079	85	4	11	13	30	57
S13	40753.1	245.4	0.1568	76	18	7	7	63	30
S14	41031.1	243.7	0.1822	65	28	7	5	73	22
S15	41209.7	242.7	0.0095	80	11	9	19	50	31
S16	41261.5	242.4	0.0097	83	10	7	6	83	10
S17	41571.2	240.6	0.1233	82	8	10	30	24	46
S18	42047.1	237.8	0.0266	87	2	11	23	23	54
S19	42197.3	237.0	0.0259	92	4	4	8	85	8
S20	42767.1	233.8	0.0053	27	69	3	3	91	5

Table S5. Characteristics of $S_0 \rightarrow T_x$ vertical transitions and fragments contributions to the GS and ES electron density for complex **2**.

			GS (hole)			ES (electron)		
state	E, cm^{-1}	λ, nm	M	L	Pz	M	L	Pz
T1	29345.9	340.8	17	81	2	1	98	1
T2	29517.3	338.8	16	82	2	1	98	1
T3	32789.1	305.0	62	30	8	4	94	2
T4	32900.4	303.9	15	82	2	1	98	1
T5	33444.2	299.0	71	20	10	5	92	3
T6	34235.8	292.1	18	80	2	2	98	1
T7	34981.7	285.9	55	37	8	5	91	4
T8	35450.2	282.1	70	19	11	13	71	16
T9	35834.5	279.1	73	7	20	27	28	44
T10	36211.5	276.2	61	4	35	29	5	65
T11	37125.4	269.4	44	3	53	8	7	85
T12	37351.6	267.7	76	2	22	43	6	51

T13	37601.5	265.9	56	2	42	34	9	57
T14	37798.7	264.6	73	3	24	32	15	53
T15	37845.5	264.2	62	7	31	18	28	54
T16	38286.2	261.2	65	7	29	15	49	36
T17	38326.6	260.9	51	8	41	15	38	47
T18	38601.9	259.1	78	3	19	10	67	23
T19	38888.6	257.1	52	4	44	19	9	72
T20	39096.2	255.8	62	5	34	17	12	71

Table S6. Characteristics of $S_0 \rightarrow S_x$ vertical transitions and fragments contributions to the GS and ES electron density for complex **3**.

					GS (hole)			ES (electron)	
state	E, cm^{-1}	λ, nm	f	M	L	Pz	M	L	Pz
S1	39370.7	254.0	0.9648	1	99	0	0	99	0
S2	39482.9	253.3	0.9498	1	99	0	0	99	0
S3	40585.9	246.4	0.0030	66	7	27	87	6	7
S4	40711.9	245.6	0.0019	66	7	27	87	6	7
S5	41063.3	243.5	0.0232	66	8	27	86	7	7
S6	41282.8	242.2	0.0011	66	3	31	87	6	7
S7	42119.3	237.4	0.1726	3	93	4	2	98	0
S8	42158.1	237.2	0.1183	4	93	3	4	96	1
S9	42917.7	233.0	0.0014	64	19	17	67	27	6
S10	43088.5	232.1	0.1154	10	85	5	10	89	1
S11	43195.7	231.5	0.1163	13	82	5	13	85	2
S12	43625.4	229.2	0.1383	34	57	9	7	92	1
S13	43842.6	228.1	0.0578	53	31	16	47	48	4
S14	43903.6	227.8	0.0354	34	56	9	26	72	3
S15	43936.0	227.6	0.0416	62	21	17	66	28	6
S16	44303.4	225.7	0.0938	25	65	11	4	95	1
S17	44467.2	224.9	0.0349	51	28	21	12	86	2
S18	44591.6	224.3	0.0526	36	51	14	2	97	1
S19	44885.2	222.8	0.0225	51	28	21	6	93	1
S20	45361.1	220.5	0.0015	75	2	23	86	6	8

Table S7. Characteristics of $S_0 \rightarrow T_x$ vertical transitions and fragments contributions to the GS and ES electron density for complex **3**.

				GS (hole)			ES (electron)	
state	E, cm^{-1}	λ, nm	M	L	Pz	M	L	Pz
T1	30152.8	331.6	1	99	0	1	99	0
T2	30263.9	330.4	1	99	0	1	99	1
T3	30390.7	329.0	1	99	0	1	99	1
T4	30457.1	328.3	1	98	1	1	99	1
T5	33173.1	301.4	0	100	0	0	100	0
T6	33215.4	301.1	0	99	1	0	100	0
T7	34688.8	288.3	1	99	0	0	99	0
T8	34711.2	288.1	0	100	0	0	100	0
T9	38776.7	257.9	66	7	26	87	6	8

T10	38935.7	256.8	67	7	27	87	6	8
T11	39218.3	255.0	67	7	26	86	6	8
T12	39552.5	252.8	62	9	29	81	11	8
T13	39674.2	252.1	6	74	20	9	73	18
T14	39691.6	251.9	3	94	4	4	93	3
T15	39736.4	251.7	5	18	77	7	17	77
T16	39809.7	251.2	5	6	89	8	4	88
T17	39822.1	251.1	3	5	92	5	3	92
T18	39919.7	250.5	2	71	27	4	69	27
T19	39929.7	250.4	2	91	7	3	91	6
T20	39997.6	250.0	2	32	66	4	29	67

Table S8. Characteristics of $S_0 \rightarrow S_x$ vertical transitions and fragments contributions to the GS and ES electron density for complex **4.**

				GS (hole)			ES (electron)		
state	E, cm^{-1}	λ, nm	f	M	L	Pz	M	L	Pz
S1	39578.2	252.7	0.8860	1	98	1	1	99	0
S2	42303.3	236.4	0.1381	2	97	1	1	99	1
S3	43238.2	231.3	0.1100	8	90	2	3	96	1
S4	43611.7	229.3	0.1485	14	78	7	2	98	0
S5	44788.6	223.3	0.0432	55	25	20	43	50	7
S6	44839.4	223.0	0.0256	55	25	20	33	61	6
S7	46196.2	216.5	0.0070	61	6	33	64	20	16
S8	46237.2	216.3	0.0111	52	28	20	14	83	3
S9	47175.6	212.0	0.0022	69	9	22	77	7	17
S10	48333.5	206.9	0.1330	62	5	33	40	8	52
S11	48746.4	205.1	0.0023	59	10	30	6	89	5
S12	49113.7	203.6	0.0151	56	13	31	35	17	49
S13	49224.3	203.2	0.0437	55	11	34	23	16	60
S14	49494.2	202.0	0.8103	29	59	13	23	66	11
S15	49762.1	201.0	0.0552	48	22	29	20	44	37
S16	49813.4	200.7	0.1236	46	29	24	8	71	21
S17	49917.5	200.3	0.0358	55	9	37	21	20	60
S18	50679.8	197.3	0.2763	52	18	30	25	66	9
S19	50923.5	196.4	0.0798	58	12	30	55	21	24
S20	51126.7	195.6	0.1337	62	12	26	62	11	27

Table S9. Characteristics of $S_0 \rightarrow T_x$ vertical transitions and fragments contributions to the GS and ES electron density for complex **4**.

			GS (hole)			ES (electron)		
state	E, cm^{-1}	λ, nm	M	L	Pz	M	L	Pz
T1	30254.8	330.5	1	98	1	1	99	0
T2	30402.0	328.9	1	98	1	1	98	1
T3	33123.0	301.9	0	100	0	0	99	0
T4	34455.7	290.2	0	100	0	0	100	0

T5	39351.8	254.1	3	0	97	5	1	94
T6	39450.6	253.5	2	0	98	5	0	95
T7	39695.9	251.9	3	36	62	4	35	61
T8	39722.1	251.7	4	14	82	5	14	81
T9	39845.6	251.0	3	55	42	4	55	42
T10	39993.6	250.0	3	95	3	2	95	2
T11	41064.1	243.5	59	11	30	69	12	19
T12	41501.5	241.0	17	2	81	20	2	79
T13	41768.6	239.4	6	28	66	5	30	65
T14	41926.1	238.5	12	50	38	13	51	36
T15	41964.7	238.3	10	80	10	10	82	8
T16	42196.0	237.0	16	15	68	17	15	67
T17	42318.6	236.3	12	24	63	14	24	62
T18	42451.9	235.6	8	77	15	9	78	13
T19	42648.2	234.5	14	45	41	15	46	39
T20	42654.9	234.4	18	58	24	20	62	19

Table S10. XYZ coordinates of all computes species.

syn-L, GS, E=-837.81644508; H=-837.52448731; G=-837.58346169

7	7.349144000	10.808746000	9.310244000
7	6.849280000	13.767830000	7.522212000
7	9.178415000	11.982886000	8.768347000
6	4.679928000	13.946866000	4.060186000
1	4.107915000	13.934395000	3.139796000
7	6.575617000	11.823519000	6.443139000
6	4.859723000	15.168006000	4.734411000
1	4.426349000	16.069365000	4.317149000
6	5.214452000	12.770725000	4.546357000
1	5.077941000	11.828070000	4.029812000
6	6.108039000	14.053535000	6.398113000
6	8.396687000	10.421152000	10.125944000
6	7.093567000	12.423874000	7.482595000
6	9.548454000	11.149545000	9.799121000
6	5.941268000	12.829389000	5.736780000
6	9.633884000	9.288581000	11.810537000
1	9.697557000	8.560285000	12.610385000
6	10.781745000	10.025883000	11.467678000
1	11.703163000	9.849884000	12.010153000
6	7.856894000	11.727917000	8.531428000
6	7.209955000	14.716822000	8.557922000
1	7.949910000	15.426113000	8.185592000
1	7.623163000	14.182572000	9.410669000
1	6.321093000	15.256703000	8.883173000
6	8.436046000	9.473201000	11.150090000
1	7.550926000	8.905292000	11.411202000
6	10.762109000	10.967794000	10.458076000
1	11.646091000	11.536273000	10.195854000
6	10.058800000	12.898810000	8.068290000
1	10.338852000	13.728552000	8.718303000
1	9.552376000	13.286735000	7.187046000
1	10.955412000	12.369054000	7.748124000
6	5.575133000	15.245708000	5.913245000
1	5.712690000	16.187330000	6.430822000

syn-L, S1, E=-837.66218379; H=-837.37356276; G=-837.43212155

7	7.538361000	10.531977000	8.961620000
7	7.126264000	13.837101000	7.304224000
7	9.089841000	12.212743000	9.049081000
6	4.374431000	13.820329000	4.270279000
1	3.641376000	13.777413000	3.473040000
7	6.168574000	11.823816000	6.779504000
6	4.938124000	15.073370000	4.601799000
1	4.624483000	15.952278000	4.050655000
6	4.729041000	12.666197000	4.929072000
1	4.289221000	11.707100000	4.684776000
6	6.260066000	14.044674000	6.269428000
6	8.464526000	10.229102000	9.870135000
6	7.081993000	12.460938000	7.584933000
6	9.462529000	11.256083000	9.948159000
6	5.687979000	12.764615000	5.967880000
6	9.688907000	8.998865000	11.515779000
1	9.814941000	8.131089000	12.153007000
6	10.669096000	10.016256000	11.558861000
1	11.517878000	9.899125000	12.222615000
6	7.890833000	11.765512000	8.469883000
6	7.509814000	14.902808000	8.216836000
1	8.372080000	15.455882000	7.844259000
1	7.743548000	14.481139000	9.191503000
1	6.668941000	15.588400000	8.331980000
6	8.589755000	9.082594000	10.693499000
1	7.842744000	8.299510000	10.652582000
6	10.574253000	11.157675000	10.773349000
1	11.326065000	11.935956000	10.813178000
6	10.035755000	13.165471000	8.491107000
1	10.149750000	14.035045000	9.138810000
1	9.695713000	13.486497000	7.510080000
1	11.004621000	12.677480000	8.374389000
6	5.883949000	15.206432000	5.610179000
1	6.317123000	16.167942000	5.856313000

syn-L, T1, E=-837.69952573; H= -837.41194060; G= -837.47065285

7	7.533613000	10.542557000	8.976955000
7	7.132208000	13.849013000	7.312481000
7	9.091154000	12.224290000	9.054314000
6	4.380240000	13.818738000	4.259134000
1	3.649137000	13.767435000	3.460961000
7	6.158600000	11.834731000	6.792505000
6	4.948063000	15.064245000	4.580719000
1	4.643804000	15.941891000	4.022195000
6	4.731112000	12.671833000	4.937702000
1	4.290141000	11.710321000	4.704565000
6	6.261248000	14.051993000	6.268730000
6	8.470299000	10.239723000	9.884490000
6	7.074928000	12.480984000	7.608449000
6	9.466978000	11.255768000	9.952733000
6	5.689003000	12.780148000	5.971357000
6	9.691105000	8.992514000	11.519349000
1	9.810486000	8.122263000	12.153911000
6	10.673491000	9.997907000	11.552641000
1	11.527451000	9.878284000	12.209335000
6	7.896918000	11.779106000	8.474601000
6	7.479034000	14.913670000	8.240502000
1	8.264893000	15.549453000	7.832250000
1	7.820045000	14.483619000	9.178212000
1	6.594993000	15.520855000	8.442875000
6	8.588405000	9.091825000	10.698861000
1	7.832724000	8.316809000	10.659051000
6	10.579528000	11.145982000	10.765775000
1	11.340734000	11.915176000	10.801518000
6	10.046640000	13.152130000	8.471216000
1	10.221212000	14.003757000	9.129287000
1	9.671875000	13.510165000	7.516392000
1	10.991657000	12.635440000	8.297040000
6	5.894996000	15.202228000	5.597054000
1	6.325861000	16.166557000	5.835633000

anti-L, GS, E=-837.81863945 ; H=-837.52662550; G=-837.58510934

7	8.833438000	12.344629000	9.246921000
7	6.642738000	13.673219000	7.600808000
7	7.848881000	10.408061000	8.696984000
6	5.205005000	14.085540000	3.792843000
1	4.846492000	14.141127000	2.771687000
7	7.046224000	11.960710000	6.212703000
6	4.953034000	15.161233000	4.663683000
1	4.407620000	16.021771000	4.294266000
6	5.896093000	12.966735000	4.211873000
1	6.091435000	12.136920000	3.542990000
6	6.085551000	14.015974000	6.389661000
6	9.353249000	11.335355000	10.031620000
6	7.204658000	12.441413000	7.422070000
6	8.750490000	10.115532000	9.695433000
6	6.341817000	12.935209000	5.534745000
6	10.665627000	10.205734000	11.660025000
1	11.419939000	10.210451000	12.437965000
6	10.050744000	8.989088000	11.312676000
1	10.345313000	8.083634000	11.830086000
6	7.956589000	11.751126000	8.473983000
6	6.595591000	14.490676000	8.799781000
1	6.810350000	13.873471000	9.666581000
1	5.597802000	14.916844000	8.897459000
1	7.331857000	15.292916000	8.743230000
6	10.328188000	11.386190000	11.030037000
1	10.799261000	12.325691000	11.293882000
6	9.085141000	8.919508000	10.327856000
1	8.618953000	7.979433000	10.059246000
6	6.963239000	9.446255000	8.065333000
1	7.493994000	8.883822000	7.296780000
1	6.127610000	9.967475000	7.608294000
1	6.587960000	8.762008000	8.825155000
6	5.386458000	15.148040000	5.974724000
1	5.194413000	15.979131000	6.642343000

anti-L, S1, E=-837.67147531; H=-837.38320243; G=-837.44267159

7	8.383604000	12.320500000	9.592346000
7	6.761129000	13.778211000	7.582194000
7	7.975413000	10.326047000	8.558764000
6	4.814719000	13.983781000	3.992833000
1	4.295926000	13.998521000	3.041310000
7	6.465413000	11.814134000	6.456015000
6	5.008989000	15.207938000	4.674943000
1	4.639016000	16.122153000	4.225712000
6	5.264484000	12.787537000	4.502741000
1	5.115795000	11.849777000	3.981861000
6	6.117738000	14.063834000	6.424696000
6	8.998921000	11.345312000	10.259221000
6	6.998154000	12.387833000	7.584279000
6	8.760720000	10.076439000	9.633560000
6	5.940132000	12.811451000	5.746456000
6	10.318424000	10.221425000	11.905310000
1	10.940189000	10.232692000	12.793087000
6	10.061368000	8.979876000	11.276953000
1	10.489336000	8.079727000	11.702302000
6	7.751172000	11.718541000	8.533121000
6	7.068245000	14.782485000	8.583617000
1	7.225468000	14.302391000	9.541519000
1	6.228535000	15.475257000	8.647529000
1	7.970475000	15.334660000	8.311572000
6	9.804694000	11.402676000	11.421463000
1	9.999656000	12.353067000	11.902816000
6	9.281810000	8.885484000	10.134557000
1	9.092903000	7.933749000	9.654382000
6	7.493448000	9.273953000	7.683009000
1	8.333641000	8.817149000	7.155144000
1	6.803514000	9.696750000	6.963054000
1	6.990815000	8.511005000	8.280658000
6	5.660298000	15.269576000	5.897134000
1	5.811612000	16.210991000	6.409566000

anti-L, T1, E=-837.70595012; H= -837.41887196; G= -837.47893438

7	8.378905000	12.315732000	9.636897000
7	6.765158000	13.787241000	7.589783000
7	7.937190000	10.333144000	8.582670000
6	4.911304000	13.963824000	3.936008000
1	4.426037000	13.967268000	2.967131000
7	6.552380000	11.826179000	6.432410000
6	5.042358000	15.180792000	4.634475000
1	4.656532000	16.088594000	4.185537000
6	5.384741000	12.781162000	4.456395000
1	5.287014000	11.842199000	3.925650000
6	6.135870000	14.064128000	6.413282000
6	9.011531000	11.328089000	10.268857000
6	7.040212000	12.410859000	7.589530000
6	8.760074000	10.069355000	9.637882000
6	6.015665000	12.818565000	5.722949000
6	10.366700000	10.207389000	11.890989000
1	11.001173000	10.218098000	12.769362000
6	10.108757000	8.977771000	11.252610000
1	10.551934000	8.074045000	11.654187000
6	7.713545000	11.719114000	8.577806000
6	7.022727000	14.785732000	8.608291000
1	7.180944000	14.298009000	9.562665000
1	6.159979000	15.449195000	8.674146000
1	7.909337000	15.373044000	8.360686000
6	9.832162000	11.384070000	11.420609000
1	10.022082000	12.334552000	11.904008000
6	9.303937000	8.888076000	10.120360000
1	9.123353000	7.934295000	9.641778000
6	7.328803000	9.320735000	7.741911000
1	7.753543000	9.331092000	6.739600000
1	6.256462000	9.486223000	7.671038000
1	7.506875000	8.349675000	8.198781000
6	5.657988000	15.251920000	5.881945000
1	5.760484000	16.194755000	6.403495000

Cu4Pz4L2 (1), E=-11920.71487534; H=-11919.77801939; G=-11919.97052341

29	7.654210000	10.085871000	10.367244000
29	6.735926000	7.506534000	12.292535000
29	9.763345000	7.638037000	11.186492000
29	8.786499000	9.882360000	13.447015000
9	4.170439000	13.258539000	10.708652000
9	4.571769000	4.503826000	9.398489000
9	8.621500000	9.705289000	17.574948000
9	5.280951000	11.808262000	9.558424000
9	4.544688000	5.800702000	11.126381000
9	10.183662000	9.684054000	16.101366000
9	10.090675000	8.145315000	17.614884000
9	6.070612000	13.779549000	9.857332000
9	6.809873000	11.091170000	15.852687000
9	5.252223000	3.781299000	11.298946000
7	9.543977000	10.138752000	9.817985000
7	10.400127000	9.163017000	10.100716000
7	3.520039000	9.847805000	11.044192000
9	10.246082000	13.034197000	7.852752000
9	13.885655000	9.270684000	9.874691000
6	3.393981000	10.100870000	12.397173000
7	8.225549000	8.392537000	14.644449000
9	6.338488000	4.492471000	15.787391000
7	7.263231000	6.239460000	10.874011000
7	11.784894000	7.280677000	14.792507000
9	6.483260000	13.216053000	15.765839000
7	7.425861000	7.460606000	14.135011000
9	8.445358000	12.398873000	15.401807000
7	6.436790000	8.910704000	9.317350000
6	11.586587000	9.478854000	9.564405000
7	4.590910000	7.817033000	8.707230000
9	10.345450000	6.651826000	8.182251000
6	7.868086000	6.859183000	16.248349000
9	9.786451000	4.681905000	7.517243000
9	12.640049000	7.681390000	10.641253000
9	5.005817000	5.799562000	14.709434000
9	6.557833000	4.760645000	13.662387000
7	10.715023000	10.141411000	13.042041000
6	11.671690000	9.261849000	13.246320000
7	8.488074000	6.377155000	10.375541000
7	7.442512000	11.234212000	12.956251000
7	12.906345000	9.735365000	12.939425000
9	12.964389000	7.882248000	8.528522000
9	9.050404000	13.065226000	9.639306000
6	12.725940000	11.030174000	12.488067000
6	4.447397000	9.406410000	13.018500000
6	5.247146000	4.849619000	10.501495000
6	7.828701000	7.453875000	6.198714000
6	5.193207000	8.613635000	9.627424000
6	7.857203000	8.224185000	7.349591000
6	9.602380000	4.840308000	13.396109000
6	9.619996000	3.817753000	14.330431000
6	8.509016000	8.052109000	15.909895000
6	4.606894000	9.046272000	10.904141000
6	6.622197000	5.305931000	10.155292000
6	11.168095000	6.043641000	14.830292000
6	5.399050000	12.794183000	10.455277000
7	6.981432000	11.364535000	11.715793000
7	10.539826000	7.138631000	12.947007000
6	11.197352000	5.010008000	15.774032000
6	10.388588000	5.967810000	13.662706000
6	2.486000000	10.882683000	13.121304000
6	6.059730000	12.337667000	11.711419000
6	13.623585000	11.990422000	12.006986000
7	5.182271000	8.751762000	12.049705000
6	10.407828000	3.901653000	15.501273000
6	7.136696000	12.206398000	15.194787000
6	11.342396000	11.270121000	12.556118000
6	5.533666000	7.573877000	7.725142000
6	10.801072000	12.488940000	12.130285000
6	11.683393000	13.440557000	11.645096000
6	7.199322000	6.530999000	15.073780000
6	13.074719000	13.194280000	11.587151000
6	7.455146000	4.810541000	9.151287000
6	12.765032000	8.571441000	9.651390000
6	11.508916000	10.702966000	8.897955000
6	8.626284000	5.538806000	9.339862000
6	10.183059000	11.072724000	9.098369000
6	2.682362000	10.946446000	14.493735000
6	3.740938000	10.255880000	15.126852000
6	5.496030000	6.800693000	6.558374000
6	4.630302000	9.475897000	14.405803000
9	10.892954000	4.942269000	9.348727000
6	6.664167000	6.752005000	5.810265000
6	6.277883000	5.389816000	14.806023000
6	5.914595000	12.874204000	12.991839000
6	11.363126000	7.884015000	13.652204000
6	9.914827000	5.457009000	8.592472000
6	6.688977000	8.270441000	8.121018000
6	6.819353000	12.126255000	13.739463000
9	8.368620000	11.972468000	7.926844000
6	9.349656000	8.904820000	16.796760000
6	9.462300000	12.291451000	8.631573000
1	2.007678000	11.549967000	15.099954000
1	1.673836000	11.419926000	12.634226000
1	3.867056000	10.352756000	16.204214000
1	5.465075000	8.962804000	14.877003000
1	4.602950000	6.255337000	6.257544000
1	6.685205000	6.156072000	4.898399000
1	8.724791000	7.376683000	5.584486000
1	8.749769000	8.752874000	7.676704000
1	7.239693000	4.057133000	8.402697000
1	8.977101000	4.809375000	12.506595000
1	9.002692000	2.934966000	14.170068000
1	10.390391000	3.074456000	16.210068000
1	11.798521000	5.074539000	16.679479000
1	7.895339000	6.316325000	17.185900000
1	5.256654000	13.669240000	13.322391000
1	12.288942000	11.236397000	8.367231000
1	14.694095000	11.800105000	11.951116000
1	13.733797000	13.969503000	11.197797000
1	11.298359000	14.392778000	11.282886000
1	9.724215000	12.643659000	12.152259000
6	3.234550000	7.300597000	8.731328000
1	2.597616000	7.857788000	8.036213000

1	3.248419000	6.244406000	8.453883000	1	12.253656000	7.657251000	16.787744000
1	2.831228000	7.378787000	9.743770000	1	13.663138000	7.320886000	15.737721000
6	2.673316000	10.393671000	9.999857000	1	12.807596000	8.888188000	15.644389000
1	3.149866000	10.245878000	9.028359000	6	14.169289000	9.023504000	13.012338000
1	1.692892000	9.906090000	10.006385000	1	14.759728000	9.367913000	13.867879000
1	2.552509000	11.467997000	10.160218000	1	13.977489000	7.951975000	13.107370000
6	12.686745000	7.813287000	15.797300000	1	14.726970000	9.187684000	12.087776000

Cu4Pz4L2 with S4 symmetry (1), E=-11920.71430051; H=-11919.77776817; G=-11919.97154329

29	1.334759000	1.191476000	1.043567000
9	-1.299653000	4.949455000	-2.663578000
9	-1.191599000	3.005337000	-3.562621000
9	0.163808000	4.569635000	-4.184084000
7	1.313617000	2.532714000	-0.386691000
7	-1.075298000	1.229869000	4.448992000
9	4.224849000	4.597885000	-0.419691000
7	0.292873000	2.613394000	-1.235344000
9	4.140347000	2.561040000	0.275259000
6	0.542598000	3.608464000	-2.098635000
7	0.059106000	1.532669000	2.552686000
9	3.113112000	4.097763000	1.357730000
6	-0.239058000	0.695625000	3.521734000
6	-1.487325000	4.908686000	2.666400000
1	-1.570855000	5.859316000	2.141606000
6	2.209441000	3.476821000	-0.706796000
6	3.429836000	3.679123000	0.125017000
6	-1.625284000	0.590844000	5.630165000
1	-2.709629000	0.727451000	5.642647000
1	-1.188191000	1.022186000	6.536892000
1	-1.416586000	-0.480700000	5.596935000
6	-1.346095000	2.519614000	4.032970000
6	-0.451427000	4.030461000	-3.125779000
6	-0.683156000	3.910445000	2.141057000
1	-0.133090000	4.033859000	1.210806000
6	-2.213992000	4.718261000	3.863829000
1	-2.836385000	5.529725000	4.239403000
6	1.766940000	4.209669000	-1.803873000
1	2.253552000	5.038501000	-2.304354000
6	-2.156200000	3.525950000	4.572033000
1	-2.715941000	3.381464000	5.494664000
6	-0.625029000	2.697481000	2.839365000
7	1.075308000	-1.229859000	4.448997000
7	-0.059094000	-1.532664000	2.552692000
6	0.239069000	-0.695617000	3.521736000
6	1.487344000	-4.908677000	2.666409000
1	1.570877000	-5.859306000	2.141616000
6	1.625290000	-0.590830000	5.630170000
1	2.709636000	-0.727435000	5.642655000
1	1.188196000	-1.022172000	6.536897000
1	1.416590000	0.480713000	5.596938000
6	1.346108000	-2.519604000	4.032977000
6	0.683174000	-3.910438000	2.141066000
1	0.133108000	-4.033854000	1.210814000
6	2.214011000	-4.718249000	3.863839000
1	2.836405000	-5.529711000	4.239413000
6	2.156215000	-3.525937000	4.572042000
1	2.715955000	-3.381449000	5.494673000
6	0.625043000	-2.697474000	2.839372000
29	-1.334750000	-1.191478000	1.043574000
9	1.299650000	-4.949418000	-2.663629000
9	1.191541000	-3.005297000	-3.562659000
9	-0.163851000	-4.569616000	-4.184100000
7	-1.313610000	-2.532724000	-0.386677000
9	-4.224801000	-4.597955000	-0.419569000
7	-0.292878000	-2.613395000	-1.235344000
9	-4.140308000	-2.561110000	0.275381000
6	-0.542612000	-3.608461000	-2.098638000
9	-3.112995000	-4.097815000	1.357803000
6	-2.209424000	-3.476846000	-0.706758000
6	-3.429782000	-3.679178000	0.125105000
6	0.451397000	-4.030437000	-3.125806000
6	-1.766941000	-4.209681000	-1.803851000
1	-2.253553000	-5.038519000	-2.304323000
29	1.191476000	-1.334756000	-1.043563000
9	4.949417000	1.299644000	2.663628000
9	3.005297000	1.191547000	3.562660000
9	4.569613000	-0.163842000	4.184113000
7	2.532719000	-1.313616000	0.386689000
7	1.229868000	1.075311000	-4.448982000
9	4.597892000	-4.224845000	0.419623000
7	2.613394000	-0.292881000	1.235352000
9	2.561060000	-4.140305000	-0.275367000
6	3.608455000	-0.542618000	2.098650000
7	1.532667000	-0.059100000	-2.552681000
9	4.097812000	-3.113038000	-1.357763000
6	0.695623000	0.239067000	-3.521727000
6	4.908679000	1.487343000	-2.666380000
1	5.859306000	1.570875000	-2.141583000
6	3.476830000	-2.209438000	0.706780000
6	3.679144000	-3.429806000	-0.125071000
6	0.590842000	1.625300000	-5.630154000
1	0.727447000	2.709646000	-5.642632000
1	1.022186000	1.188211000	-6.536882000
1	-0.480701000	1.416600000	-5.596926000
6	2.519611000	1.346109000	-4.032957000
6	4.030435000	0.451397000	3.125812000
6	3.910440000	0.683165000	-2.141046000
1	4.033855000	0.133094000	-1.210797000
6	4.718253000	2.214016000	-3.863806000
1	5.529715000	2.836416000	-4.239373000
6	4.209668000	-1.766953000	1.803872000
1	5.038500000	-2.253569000	2.304349000
6	3.525945000	2.156221000	-4.572014000
1	3.381459000	2.715967000	-5.494642000
6	2.697479000	0.625038000	-2.839355000
7	-1.229862000	-1.075290000	-4.448998000
7	-1.532666000	0.059102000	-2.552685000
6	-0.695619000	-0.239058000	-3.521731000

6	-4.908688000	-1.487317000	-2.666425000
1	-5.859320000	-1.570845000	-2.141637000
6	-0.590833000	-1.625266000	-5.630174000
1	-0.727442000	-2.709611000	-5.642666000
1	-1.022171000	-1.188164000	-6.536898000
1	0.480711000	-1.416570000	-5.596938000
6	-2.519609000	-1.346086000	-4.032983000
6	-3.910446000	-0.683154000	-2.141074000
1	-4.033864000	-0.133093000	-1.210819000
6	-4.718260000	-2.213976000	-3.863859000
1	-5.529725000	-2.836363000	-4.239441000
6	-3.525945000	-2.156183000	-4.572057000
1	-3.381457000	-2.715917000	-5.494692000
6	-2.697479000	-0.625028000	-2.839374000
29	-1.191478000	1.334751000	-1.043562000
9	-4.949398000	-1.299672000	2.663641000
9	-3.005282000	-1.191541000	3.562676000
9	-4.569617000	0.163835000	4.184111000
7	-2.532724000	1.313605000	0.386689000
9	-4.598009000	4.224755000	0.419531000
7	-2.613391000	0.292872000	1.235357000
9	-2.561158000	4.140294000	-0.275408000
6	-3.608459000	0.542602000	2.098650000
9	-4.097835000	3.112928000	-1.357817000
6	-3.476859000	2.209407000	0.706760000
6	-3.679212000	3.429747000	-0.125123000
6	-4.030428000	-0.451409000	3.125819000
6	-4.209692000	1.766924000	1.803855000
1	-5.038540000	2.253526000	2.304318000

Cu4Pz4L1 (2), E=-11083.66278829; H=-11083.02143370
; G=-11083.18456561

29	20.943188000	0.957267000	13.954979000
29	21.595572000	4.069828000	14.461158000
29	18.844764000	2.775814000	12.994912000
29	18.916099000	4.593039000	15.551830000
9	22.910315000	4.918277000	17.255477000
9	20.849306000	5.121939000	17.812047000
9	18.488308000	3.193094000	9.732151000
9	22.221943000	4.167724000	19.158376000
9	16.487648000	-1.059132000	14.553293000
9	18.439497000	-0.752940000	13.715004000
9	18.712117000	3.338446000	18.571902000
9	22.721585000	-1.032023000	12.517359000
9	21.277458000	-2.432704000	11.777230000
9	16.757609000	0.302751000	12.903286000
9	17.234885000	1.455069000	9.737406000
9	16.679704000	3.868147000	18.145894000
9	22.697144000	-1.557355000	10.423135000
9	17.125174000	1.959238000	19.039251000
7	19.596623000	1.426474000	11.658420000
7	20.514462000	0.605834000	12.166786000
7	21.562749000	2.793373000	15.942961000
7	18.028349000	2.149243000	14.652210000
9	18.740508000	1.682629000	8.215713000
7	18.105714000	2.920849000	15.741802000
7	21.263399000	1.512803000	15.708657000
7	20.195040000	6.224100000	11.151519000
7	21.052824000	5.993288000	14.647782000
7	22.060795000	3.620572000	12.581747000
7	19.186730000	4.580675000	12.268266000
7	19.844036000	6.176341000	15.178652000
9	21.943428000	-1.290359000	16.635756000
7	21.956517000	3.576120000	10.355642000
9	20.451397000	-0.949910000	18.149814000
9	19.905801000	-0.953776000	16.064827000
6	20.267555000	4.950001000	11.613918000
9	18.460253000	8.278199000	17.035841000
6	21.443814000	4.071865000	11.510418000
6	18.966631000	6.716509000	11.550981000
6	23.044651000	2.771121000	12.120435000
6	20.974674000	-0.192002000	11.194342000
6	21.112881000	0.882969000	16.881929000
6	21.901108000	4.302254000	17.872962000
9	23.035666000	8.531248000	13.127532000
6	18.341295000	5.669694000	12.248153000
6	21.617936000	2.964570000	17.269507000
6	21.338932000	1.769277000	17.929017000
1	21.298306000	1.580119000	18.994720000
6	22.987163000	2.728509000	10.717614000
6	17.300451000	-0.138046000	14.038403000
6	17.649148000	2.227489000	16.793628000
6	17.509214000	0.971033000	15.019531000
6	17.070376000	5.836656000	12.811510000
1	16.604185000	5.020058000	13.361978000
6	23.977306000	2.001851000	12.827688000
1	24.003373000	2.035796000	13.916285000
6	19.476490000	1.153543000	10.356047000
6	21.929495000	-1.304361000	11.480741000
9	23.830717000	7.164904000	14.591630000
6	20.334867000	0.112194000	9.995635000
1	20.467508000	-0.348424000	9.023543000
6	18.363561000	7.970295000	11.396012000
1	18.856187000	8.783962000	10.866228000
6	21.491215000	3.805171000	9.000372000
1	20.570427000	4.390991000	9.022914000
1	21.274060000	2.844738000	8.521385000
1	22.252223000	4.339399000	8.422308000
6	18.479926000	1.873751000	9.508151000
6	23.841019000	1.915468000	9.963442000
1	23.783531000	1.872834000	8.877102000
9	17.278182000	7.134605000	15.661808000
6	21.512494000	7.184694000	14.252436000
9	23.116424000	6.418005000	12.715397000
6	17.108725000	8.127054000	11.967687000
1	16.603219000	9.088249000	11.883906000
6	19.534581000	7.478863000	15.121462000
6	17.244956000	0.960425000	16.387584000
1	16.823274000	0.162948000	16.987074000
6	16.468231000	7.074746000	12.660874000
1	15.487414000	7.250752000	13.100041000
6	24.827649000	1.198315000	12.086108000
1	25.558448000	0.574417000	12.598685000
6	21.210070000	6.979352000	10.441123000
1	21.423638000	7.904941000	10.985307000
1	20.866243000	7.218819000	9.429601000
1	22.130511000	6.395628000	10.385392000
6	24.756580000	1.153379000	10.675172000
1	25.435591000	0.497888000	10.131570000
6	20.847876000	-0.586234000	16.934321000
6	17.545461000	2.851441000	18.147215000
6	22.879877000	7.324475000	13.670365000
6	18.282211000	8.008452000	15.739074000
9	17.900247000	9.137787000	15.147895000
6	20.581290000	8.185680000	14.537108000
1	20.655403000	9.250648000	14.351018000

Ag4Pz4L2 (3), E=-26783.43245433; H=-26782.49707379; G=-26782.69606477

47	7.698760000	10.269933000	10.328614000
47	6.598982000	7.395405000	12.398274000
47	9.865845000	7.544218000	11.033484000
47	8.696800000	9.903845000	13.706593000
9	4.023531000	13.413384000	10.712271000
9	4.348750000	4.443407000	9.298789000
9	8.737532000	9.338005000	17.837244000
9	5.271665000	12.031511000	9.619923000
9	4.416770000	5.834816000	10.951303000
9	10.261614000	9.339128000	16.327674000
9	10.233231000	7.793680000	17.837441000
9	5.961825000	14.022769000	10.011224000
9	6.537828000	11.113010000	15.946290000
9	5.002715000	3.793957000	11.240056000
7	9.787452000	10.285545000	9.606915000
7	10.636825000	9.308654000	9.894021000
7	3.385359000	10.011471000	10.854854000
9	10.500391000	13.258114000	7.777808000
9	14.147404000	9.414553000	9.732974000
6	3.213580000	10.318800000	12.192172000
7	8.176009000	8.133958000	14.985403000
9	6.595953000	4.035650000	15.877389000
7	7.172338000	6.001317000	10.750437000
7	12.009093000	7.334954000	14.903225000
9	5.832717000	13.148098000	15.952565000
7	7.388663000	7.207266000	14.455716000
9	7.930998000	12.717608000	15.688805000
7	6.293274000	8.861291000	9.238152000
6	11.847430000	9.662960000	9.446860000
7	4.405368000	7.831209000	8.651138000
9	10.235852000	6.374949000	8.068535000
6	8.073640000	6.426342000	16.441966000
9	9.540724000	4.501027000	7.258823000
9	12.879568000	7.876375000	10.563178000
9	5.182469000	5.304406000	14.852086000
9	6.829307000	4.458673000	13.777553000
7	10.877257000	10.150962000	13.117862000
6	11.833703000	9.268163000	13.304408000
7	8.390749000	6.087727000	10.234071000
7	7.221288000	11.462929000	13.131347000
7	13.061915000	9.731671000	12.954862000
9	13.168651000	8.005037000	8.441494000
9	9.317898000	13.224978000	9.575280000
6	12.874977000	11.020939000	12.491904000
6	4.209052000	9.602276000	12.879588000
6	5.055550000	4.806224000	10.372007000
6	7.643398000	7.230062000	6.188875000
6	5.035683000	8.635784000	9.546964000
6	7.699678000	8.034637000	7.315162000
6	9.893828000	4.785762000	13.600109000
6	9.967017000	3.785193000	14.555601000
6	8.604771000	7.688911000	16.172756000
6	4.440513000	9.157440000	10.789350000
6	6.453934000	5.159962000	9.997613000
6	11.433495000	6.080577000	14.983275000
6	5.283285000	13.003468000	10.543199000
7	6.850088000	11.630695000	11.867666000
7	10.737895000	7.105986000	13.085011000
6	11.517047000	5.070333000	15.948708000
6	10.638880000	5.949707000	13.830508000
6	2.310954000	11.159182000	12.855401000
6	5.871521000	12.543437000	11.833413000
6	13.766756000	11.971109000	11.981007000
7	4.953495000	8.884421000	11.968347000
6	10.766965000	3.927230000	15.712557000
6	6.692509000	12.313960000	15.376139000
6	11.494959000	11.269908000	12.599138000
6	5.345001000	7.505374000	7.690511000
6	10.952933000	12.493646000	12.184407000
6	11.829649000	13.435526000	11.670282000
6	7.311156000	6.174986000	15.304962000
6	13.216220000	13.177333000	11.571723000
6	7.229983000	4.663401000	8.948354000
6	13.010643000	8.738141000	9.545539000
6	11.793541000	10.915580000	8.832014000
6	8.451621000	5.299446000	9.153253000
6	10.451192000	11.257114000	8.967744000
6	2.454552000	11.256271000	14.232295000
6	3.455400000	10.542623000	14.931089000
6	5.280655000	6.695970000	6.549815000
6	4.340008000	9.705933000	14.270532000
9	10.670255000	4.545284000	9.093236000
6	6.450326000	6.570331000	5.813736000
6	6.480621000	4.987194000	14.956578000
6	5.592989000	13.005062000	13.120392000
6	11.546856000	7.896058000	13.755434000
6	9.724447000	5.179161000	8.386892000
6	6.527664000	8.162783000	8.072956000
6	6.483467000	12.273674000	13.900790000
9	8.626688000	12.184442000	7.832606000
6	9.458833000	8.540529000	17.045776000
6	9.728310000	12.487239000	8.535994000
1	1.781322000	11.904137000	14.792585000
1	1.543788000	11.716359000	12.320324000
1	3.541129000	10.668352000	16.009522000
1	5.133594000	9.170852000	14.790282000
1	4.366120000	6.181015000	6.260366000
1	6.451133000	5.944501000	4.921871000
1	8.539125000	7.089260000	5.585655000
1	8.615745000	8.532471000	7.629907000
1	6.950021000	3.969693000	8.164480000
1	9.264484000	4.702079000	12.715305000
1	9.385060000	2.874148000	14.423618000
1	10.793307000	3.116162000	16.439476000
1	12.127852000	5.178515000	16.843317000
1	8.228358000	5.794674000	17.308617000
1	4.859441000	13.737391000	13.435746000
1	12.595248000	11.479153000	8.369168000
1	14.833538000	11.771400000	11.895714000
1	13.869680000	13.945890000	11.160360000
1	11.442702000	14.390361000	11.317506000
1	9.879904000	12.670385000	12.246011000
6	3.030792000	7.365949000	8.684137000
1	2.437902000	7.870066000	7.913604000

1	3.011385000	6.286445000	8.516696000
1	2.599611000	7.566378000	9.667887000
6	2.614739000	10.557663000	9.752585000
1	3.133228000	10.358637000	8.811671000
1	1.615791000	10.110151000	9.722732000
1	2.529917000	11.640374000	9.874567000
6	12.902937000	7.921863000	15.885193000

1	12.469455000	7.809018000	16.881861000
1	13.882256000	7.432878000	15.850983000
1	13.020761000	8.988542000	15.680236000
6	14.327398000	9.021716000	12.999888000
1	14.959729000	9.413593000	13.803657000
1	14.143311000	7.957673000	13.166861000
1	14.838326000	9.134954000	12.040718000

Ag4Pz4L1 (4), E=-25946.37822658; H=-25945.73666929; G=-25945.90790866

47	3.534111000	10.441989000	6.522722000
47	6.898589000	9.666322000	6.130021000
47	5.214956000	10.337246000	9.202737000
47	6.972204000	7.495154000	8.407026000
9	1.207780000	7.112360000	4.151169000
9	1.405664000	9.559064000	8.368695000
9	9.664656000	11.375432000	5.586160000
9	1.999931000	12.726666000	5.334148000
7	4.917228000	7.290894000	8.830933000
9	0.779234000	8.397461000	10.063988000
9	10.736673000	5.621121000	8.626965000
9	1.260251000	7.272049000	6.290670000
7	8.913006000	8.063872000	7.799426000
9	2.243235000	9.964187000	10.307356000
7	4.045147000	8.627157000	5.579902000
9	1.385408000	9.047440000	5.089621000
7	7.386349000	10.900580000	9.298195000
7	6.879468000	13.860294000	7.490971000
9	11.466901000	7.082798000	10.020499000
7	9.244592000	12.035580000	8.826475000
9	0.477859000	13.187863000	6.771884000
7	4.042613000	12.265947000	8.782182000
7	4.192320000	8.372625000	9.105129000
7	8.945471000	9.003735000	6.860338000
9	7.109736000	5.862478000	6.018119000
7	3.311064000	12.193987000	7.676302000
9	6.438166000	5.157802000	4.101855000
7	5.316034000	8.241668000	5.492643000
9	10.591495000	9.824551000	4.434597000
9	3.941341000	3.913616000	9.052642000
9	7.566090000	6.991015000	4.251490000
9	1.658540000	14.774418000	5.930330000
9	5.920366000	4.745526000	8.828578000
9	9.378773000	6.529246000	10.016531000
6	4.039683000	6.559895000	4.726980000
1	3.717774000	5.606208000	4.326178000
6	4.599116000	13.839856000	4.073575000
1	3.997908000	13.775922000	3.167958000
6	10.561367000	10.382491000	5.646716000
7	6.645761000	11.887040000	6.481025000
6	11.050458000	8.680889000	7.569560000
1	12.125337000	8.756087000	7.686501000
6	3.260185000	7.642803000	5.123800000
9	4.587815000	15.147582000	10.780541000
6	3.892976000	13.489824000	9.296118000
6	4.724390000	15.087919000	4.724914000
1	4.217060000	15.955695000	4.305926000
6	2.797586000	6.613020000	9.054248000
1	1.904253000	6.001167000	9.095290000
9	11.756961000	10.926318000	5.870177000
6	5.211804000	12.700109000	4.567785000
1	5.116101000	11.732006000	4.075942000
6	6.092326000	14.080542000	6.376579000
6	1.832536000	8.980360000	9.505143000
6	2.917217000	7.988532000	9.244761000
9	4.598436000	4.633505000	7.140627000
9	5.781840000	13.353259000	10.681393000
6	4.108948000	6.224196000	8.795837000
6	8.392986000	10.437514000	10.118737000
6	5.335642000	7.002730000	4.985977000
6	7.172086000	12.535086000	7.498447000
6	9.573726000	11.141758000	9.828060000
6	10.214204000	9.391783000	6.706080000
6	5.960911000	12.831799000	5.745610000
6	1.770611000	7.766327000	5.161326000
6	10.438228000	6.771933000	9.237840000
6	9.567610000	9.179352000	11.762282000
1	9.599433000	8.403557000	12.525684000
6	10.753299000	9.883878000	11.452506000
1	11.669333000	9.634499000	11.986046000
6	7.929427000	11.841052000	8.554891000
9	3.862132000	13.306842000	11.631180000
6	4.641503000	4.870451000	8.453589000
6	7.259230000	14.871016000	8.460787000
1	7.947525000	15.591934000	8.007397000
1	7.741518000	14.396719000	9.317685000
1	6.364657000	15.393183000	8.814076000
6	8.376967000	9.442374000	11.106000000
1	7.460019000	8.898105000	11.331920000
6	10.780768000	10.878415000	10.486628000
1	11.694653000	11.419675000	10.247742000
6	4.532411000	13.828894000	10.601008000
6	10.157392000	7.853287000	8.247263000
6	10.165191000	12.961632000	8.191974000
1	10.474608000	13.733506000	8.904258000
1	9.681755000	13.430860000	7.332413000
1	11.044963000	12.416877000	7.837039000
6	2.695400000	13.366272000	7.480111000
6	3.028450000	14.250867000	8.504350000
1	2.697349000	15.272627000	8.650941000
6	6.616528000	6.252331000	4.829853000
6	1.708518000	13.521933000	6.370756000
6	5.475512000	15.235509000	5.881388000
1	5.571525000	16.197569000	6.381879000
